# Developmental Trajectory of Body Weight in Youths at Risk for Major Mood Disorders

**DOI:** 10.1001/jamanetworkopen.2023.38540

**Published:** 2023-10-19

**Authors:** Nitya Adepalli, Jill Cumby, Niamh Campbell, Barbara Pavlova, Martin Alda, Leah E. Cahill, Rudolf Uher

**Affiliations:** 1 Department of Psychiatry, Dalhousie University, Halifax, Nova Scotia, Canada; 2 Nova Scotia Health Authority, Halifax, Nova Scotia, Canada; 3 Department of Community Health and Epidemiology, Dalhousie University, Halifax, Nova Scotia, Canada

## Abstract

**Importance:**

Mood disorders are associated with increased body weight, especially in females, but it remains unknown when the weight increase starts.

**Objectives:**

To examine sex-specific weight trajectories associated with familial mood disorder risk and determine the age at which youth at familial risk for mood disorders begin to diverge in weight from controls.

**Design, Setting, and Participants:**

This community-based, single-center, acceleration cohort study of youth at familial risk for mood disorders and controls with yearly follow-ups (mean [SD], 5 [2.1] years) from January 1, 2014, to December 31, 2022, assessed 394 unaffected female and male offspring (aged 3 to 20 years) of parents with or without a mood disorder. Parents with mood (depressive or bipolar) disorders were recruited through adult mental health services. Parents of control participants were matched on age and socioeconomic factors and recruited through acquaintance referrals or schools.

**Exposures:**

The youth in the familial mood risk group had at least 1 parent with a major mood disorder, whereas control youth did not have a parent with a mood disorder.

**Main Outcomes and Measures:**

Body mass indexes (BMIs) were calculated as weight in kilograms divided by height in meters squared from measured weight and height at annual assessments and then converted to age- and sex-adjusted *z* scores (*z*BMIs). Repeated-measure regressions examined the association between *z*BMI and age in youth at familial risk of mood disorders and controls while accounting for sex. Sensitivity analyses accounted for socioeconomic status, prematurity, and birth weight.

**Results:**

Of 394 participants (mean [SD] age, 11.5 [3.6] years; 203 [51.5%] female), youths at familial risk for mood disorders showed overall no difference in body weight (β = 0.12; 95% CI, 0.01-0.24) from controls. A sex-specific difference was detected, with females at familial risk showing a rapid peripubertal increase in body weight, leading to significantly increased *z*BMIs at 12 years and older compared with controls (β = 0.57; 95% CI, 0.31-0.82) independent of socioeconomic status, prematurity, or birth weight. Males did not differ from controls at any age.

**Conclusions and Relevance:**

In this cohort study, females with a family history of mood disorders were prone to weight gain starting around puberty and predating mood disorder onset. Early interventions aiming to prevent adverse mental and physical outcomes in this vulnerable group need to start in childhood.

## Key Points

**Question:**

When do offspring with familial risk of mood disorders begin to diverge from control offspring in their body mass?

**Findings:**

In this cohort study of 394 individuals, offspring with familial mood risk overall showed no difference in body weight compared with controls. Females 12 years or older had significantly higher body mass associated with their family history of mood disorders, while males showed no difference; this association was independent of socioeconomic status, birth weight, and prematurity status.

**Meaning:**

These findings suggest that adolescents with a family history of mood disorders may be at increased risk for developing future weight issues, with females being the most vulnerable.

## Teaser

This cohort study examines whether youths at familial risk for mood disorders have increased weight compared with control offspring beginning in adolescence.

## Introduction

Major depressive disorder (MDD) and bipolar disorder (BD), jointly referred to as mood disorders, carry a significant burden of morbidity and premature mortality due to associated physical illnesses. Mood disorders typically begin in adolescence or early adulthood and affect 30% of people at some point in their lives.^1,2^ These disorders decrease expected lifespan by 9 to 16 years.^3,4,5,6^ People with mood disorders have increased body weight, which is associated with greater risk of physical illness and mortality.^7,8^ Longitudinal studies^9,10^ show a connection between adolescent obesity and adult depression and vice versa, but it is unclear when during development the connection between mood disorder risk and body weight arises.

The association between mood disorders and weight begins early and acts bidirectionally. Depressed mood is associated with increased weight in adolescents, and depressive symptoms exacerbate future weight gain.^9,11,12^ Overweight and obesity are also associated with earlier onset and increased severity of depression and BD.^13,14,15^ However, with both obesity and mood disorders being already common in adolescence and prior longitudinal studies^16,17^ not reaching back into childhood, it is unclear when the association between body weight and mood risk starts.

Children of parents with mood disorders are at increased risk of developing one themselves.^18,19,20,21^ In fact, having a parent with MDD increases risk of MDD by 2.4 times and increases risk of BD 5 times compared with individuals whose parents do not have a severe mental illness, and offspring of parents with BD are at a 4.1-fold increased risk of BD as well as a 2.1-fold increased risk of MDD.^22,23^ Studying transmission of the risk of mood disorders as a category captures this transdiagnostic risk. By following up offspring prospectively, we can examine the emergence of the association between weight and familial mood disorder risk.

The association between obesity and depression exists in both sexes but is much stronger in females.^24,25,26^ Obesity is associated with diagnosis of MDD in women but not men.^27^ Additionally, in females, depression in childhood is associated with obesity in late adolescence, and obesity in late adolescence is associated with depression in early adulthood.^28,29^

This study compared the trajectories of body mass index (BMI) during development between offspring of parents with and without mood disorders to find the age when they diverge. Given the evidence that the association between mood disorders and obesity is stronger in females, we also considered sex differences. We hypothesized that individuals at familial risk would display increased weight compared with control offspring beginning in adolescence, and we expected weight increases to be most pronounced in females with familial risk.

## Methods

### Participants

For this cohort study, we recruited children and youth with or without familial risk of mood disorders as a part of the Families Overcoming Risks and Building Opportunities for Well-being (FORBOW) project in Nova Scotia, Canada.^30^ To examine the trajectories of weight during a broad developmental period, we included participants aged 3 to 20 years. Participants were excluded if they had a lifetime diagnosis of MDD (n = 64) or BD (n = 2). Participants who could give written informed consent to participate in the study did so; those who could not provide written consent gave assent, and a legal guardian provided written informed consent on their behalf. All study procedures were approved by the Research Ethics Board of the Nova Scotia Health Authority. This study followed reporting guidelines laid out by Strengthening the Reporting of Observational Studies in Epidemiology (STROBE) for cohort studies.^31^

All participants were recruited through their parents. Parents with mood disorders were recruited by referral from the clinicians treating them. Parents of control participants were healthy individuals matched on demographic factors to affected parents and recruited through referral of acquaintances living in the same neighborhoods as affected parents or by contacting parents of children who attended the same schools.

### Parental and Offspring Diagnoses

Parents were assessed for psychiatric diagnoses by interviewers trained in the Structured Clinical Interview of the *Diagnostic and Statistical Manual of Mental Disorders* (Fifth Edition) (*DSM-5*).^32^ Parent assessors were masked to offspring diagnoses. Offspring were assigned familial high-risk status if they had at least 1 biological parent with a diagnosis of MDD or BD. Control offspring were individuals whose parents had no lifetime diagnosis of MDD or BD. Offspring were interviewed by youth assessors masked to parent diagnoses. Children younger than 18 years were assessed for *Diagnostic and Statistical Manual of Mental Disorders* (Fourth Edition) diagnoses using the Kiddie Schedule for Affective Disorders and Schizophrenia, and youth 18 years and older were assessed for *DSM-5* diagnoses using the Structured Clinical Interview of the *DSM-5*. Interviewers also gathered participant- or parent-reported ethnicity, socioeconomic characteristics, prematurity status, and birth weight. Ethnicity information was gathered to ensure there was no significant difference between control and high-risk groups because ethnicity is known to be associated with BMI.^33^ Parent and offspring diagnoses were confirmed in consensus meetings with a psychiatrist.^34^ Participants were excluded from analysis if either parent had a history of nonaffective psychotic disorders or if the participant received a diagnosis of MDD or BD.

### Physical Measurements

To capture development over time, we assessed offspring repeatedly in yearly intervals from January 1, 2014, to December 31, 2022, for a mean (SD) of 5 (2.1) years. Youth assessors measured height, weight, and waist circumference of participants using calibrated HS-250 Brecknell scales and measuring tapes. We calculated BMI as weight in kilograms divided by height in meters squared.^2^ We calculated waist-to-height ratio as waist circumference in centimeters divided by height in centimeters.^35^ We transformed BMI to age- and sex-adjusted *z* scores (*z*BMIs) based on reference data from the Centers for Disease Control and Prevention using the growthcleanr R package^28,36^; *z*BMI is the established way to quantify body mass in children and adolescents.^37^ We chose *z*BMI for the primary analyses because it provides a higher number of valid measurements. Secondary analyses using waist-to-height ratio as the dependent variable are included in eMethods 1 in Supplement 1. The BMIs were consistent for all ages and sexes in adults but not children. An adjustment based on an external reference standard is necessary to make BMIs comparable across age and sexes in children.

### Statistical Analysis

We explored the association of age with *z*BMI in individuals with and without familial mood risk, with age and familial risk status as the independent variables and *z*BMI as the dependent variable. To examine the development of *z*BMI over age without making assumptions about the shape of the trajectory, we fitted a nonparametric local-linear kernel regression with 500 bootstrap replications using the npregress package in Stata SE software, version 16 (StataCorp).^38^

Given the prior evidence of sex differences, we performed additional analyses stratified by age and sex. For these analyses, we used the age of 12 years as the cut point because depressive symptoms are known to begin with pubertal onset, and 12 years is a midpoint between average pubertal onset of girls and boys.^39,40^ In supplementary analyses, we used pubertal onset instead of the cut point of 12 years of age (eMethods 2 in Supplement 1).

To examine the contribution of nonindependence of repeated measurements of an individual, we ran sensitivity analyses using a subset that included only the most recent measurement for each person. This was done because it was not possible to include individual identifiers as a covariate with the npregress package. In primary analyses, we did not include socioeconomic status (SES) as a covariate because mental illness negatively impacts SES; individuals with severe mental illness are more likely to struggle finding jobs and to live in lower-income neighborhoods.^41,42^ However, to ensure our results were not completely explained by SES, we conducted sensitivity analyses with SES as a covariate. Socioeconomic status was measured as a sum of 5 binary indicators: increased SES was indicated by mother’s education past high school, father’s education past high school, home ownership, annual household income above $60 000, and bedrooms-to-people ratio of 1 or above.^43^

Our final sensitivity analysis included offspring birth weight and prematurity status as covariates. Higher birth weight and prematurity are associated with increased rates of overweight and obesity and are risk factors for mood disorders.^44,45,46,47^ We categorized birth weight as large (≥4.0 kg), average (2.6-3.9 kg), or low (<2.6 kg). Prematurity status was classified as extremely preterm (<28 weeks), very preterm (28 to <32 weeks), moderate to late preterm (32 to <37 weeks), or full term or overdue (≥37 weeks). Missing data on birth weight (3.2% of assessments) and prematurity (5.4% of assessments) were imputed with multivariate imputation by chained equations using script provided by Austin et al^48^ with 25 imputations. To account for the multiple tests conducted, we reported nominal significance at a 2-sided *P* < .05 as well as Bonferroni-corrected statistical significance at a 2-sided *P* < .007 after accounting for 7 comparisons.

## Results

### Demographic Characteristics

A total of 394 participants (mean [SD] age, 11.5 [3.6] years; 203 [51.5%] male and 191 [48.5%] female; 358 [90.9%] of European ethnicity and 36 [9.1%] of other ethnicity, including Asian, Black, First Nations, Hispanic, and other) were included in the study. The cohort was enriched with offspring of parents with mood disorders such that 254 individuals (64.5%) in our sample (1415 measurements of BMI from 394 offspring) had familial risk for mood disorders. Individuals with a family history of mood disorders had significantly lower SES. Demographic characteristics of participants are displayed in the Table. We also considered the demographic characteristics of individuals excluded based on availability of valid measurements (eTable 1 in Supplement 1). Of the 437 participants initially evaluated for inclusion, 43 (9.8%) had missing data on weight or height and did not contribute to analysis.

**Table.** Demographic Characteristics of Participants Who Provided Valid Height and Weight Data^a^

Characteristic	Participants with high familial risk (n = 254)	Controls (n = 140)	*P* value^b^
Sex			
Female	118 (46.5)	73 (52.1)	.28
Male	136 (53.5)	67 (47.9)	
BMI measurements			
Total	929 (100)	486 (100)	
Female participants^c^	420 (45.2)	249 (51.2)	.03
Male participants	509 (54.8)	237 (48.8)	
WtH measurement available	544 (58.6)	334 (68.7)	
Female participants	256 (47.0)	167 (50.0)	.40
Male participants	288 (53.0)	167 (50.0)	
Participants with WtH available	195 (76.8)	112 (80.0)	
SES by category^c^			
0	11(4.3)	2(1.4)	<.001
1	27 (10.6)	3 (2.1)	
2	44 (17.3)	13 (9.3)	
3	52 (20.5)	47 (33.6)	
4	86 (33.5)	52 (37.1)	
5	34 (13.4)	23 (16.4)	
Ethnicity			
European ancestry	233 (91.7)	125 (89.3)	.28
Other^d^	21 (8.3)	15 (10.7)	
Prematurity status by category^c^			
Extremely preterm	25 (9.8)	16 (11.4)	<.001
Very preterm	10 (3.9)	0	
Moderate to late preterm	1 (0.4)	1 (0.7)	
Full term or overdue	218 (85.8)	123 (87.9)	
Birth weight by category			
Low	59 (23.2)	28 (20.0)	.35
Average	177 (69.7)	106 (75.7)	
Large	18 (7.1)	6 (4.3)	
Age, mean (SD), y	11.6 (3.6)	11.3 (3.5)	.25
BMI *z* score, mean (SD)	0.34 (1.1)	0.25 (1.1)	.11
WtH *z* score, mean (SD)	0.19 (0.98)	0.09 (0.85)	.12

Abbreviations: BMI, body mass index (calculated as weight in kilograms divided by height in meters squared); SES, socioeconomic status; WtH, weight-to-height ratio.

^a^ Data are presented as number (percentage) of participants or BMI measurements unless otherwise indicated.

^b^ Differences between groups were tested using the χ^2^ test for categorical variables and the *t* test for continuous variables.

^c^ Statistically significant differences between groups at *P* < .05.

^d^ The other ethnicities reported were Asian, Black, First Nations, Hispanic, and other.

### Association of Parental Mood Disorder Diagnosis With BMI

**Figure 1.**
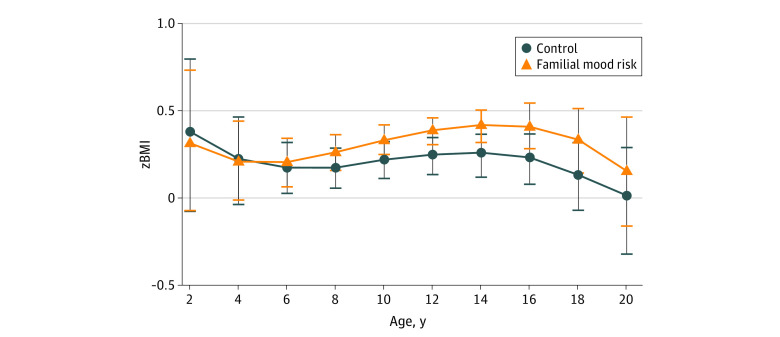
Association of Familial Mood Disorder Risk Status With Body Mass Index *z* Score (*z*BMI) The association of familial mood disorder risk status with *z*BMI in all offspring as modeled by nonparametric regression estimates. Error bars represent the SEM at each 2-year age interval.

**Figure 2.**
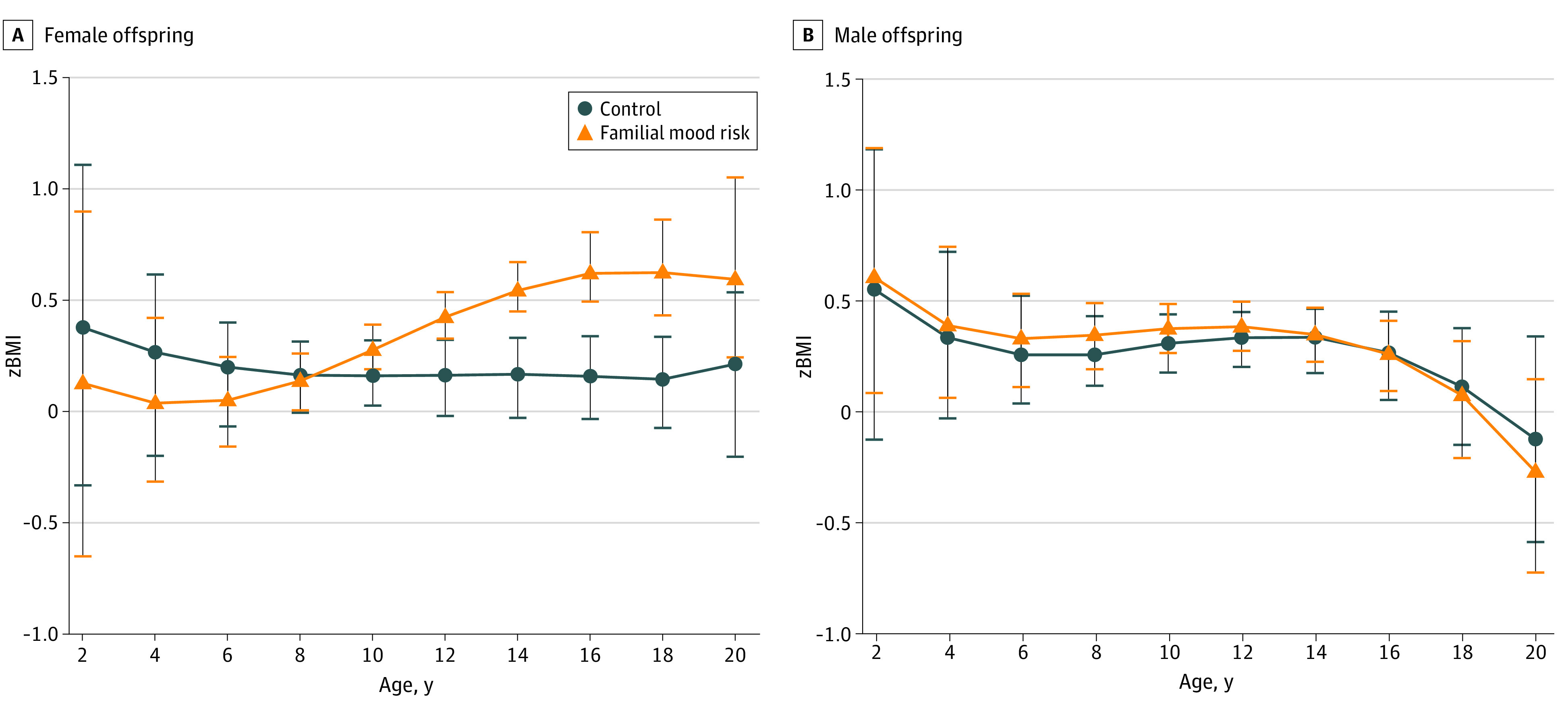
Association of Familial Mood Disorder Risk Status With Offspring Body Mass Index *z* Score (*z*BMI) by Sex The association of familial mood disorder risk status on *z*BMI in male and female offspring as modeled by nonparametric regression estimates. Error bars represent the SEM at each 2-year age interval.

For 1415 assessments in 394 participants, *z*BMI did not significantly differ between individuals at familial risk for mood disorders and controls (β = 0.12; 95% CI, 0.01-0.24; *P* = .051) (Figure 1). In sex-stratified analyses, we found that female offspring with familial mood risk had a significantly higher *z*BMI than controls (β = 0.19; 95% CI, 0.01-0.37; *P* = .049) (Figure 2A), whereas male offspring with familial mood risk did not significantly differ on *z*BMI from controls (β = 0.04; 95% CI, −0.12 to 0.21; *P* = .64) (Figure 2B).

When participants were separated into younger (3-11 years) and older (12-20 years) age groups, we found that younger female offspring at familial risk for mood disorders did not differ in *z*BMI from controls (β = −0.10; 95% CI, −0.32 to 0.12; *P* = .36), whereas older female offspring with familial risk had a significantly higher *z*BMI than controls (β = 0.57; 95% CI, 0.31 to 0.82; *P* < .001) (Figure 2A), a statistically significant difference after Bonferroni correction. Younger male offspring with familial mood risk did not differ significantly in *z*BMI from controls (β = 0.05; 95% CI, −0.14 to 0.25; *P* = .59). Older male offspring with familial mood risk also showed no difference in *z*BMI from controls (β = −0.01; 95% CI, −0.27 to 0.24, *P* = .93) (Figure 2B).

### Repeated Measurements

When only 1 *z*BMI measurement per individual was retained, *z*BMI did not differ by group in all individuals (β = 0.16; 95% CI, −0.11 to 0.40; *P* = .20) or in female offspring (β = 0.22; 95% CI, −0.15 to 0.55; *P* = .20), although effect sizes remained similar to primary results. Male offspring at familial risk also did not differ from controls (β = 0.15; 95% CI, −0.17 to 0.44; *P* = .34). After age stratification, neither younger (β = −0.04; 95% CI, −0.55 to 0.46; *P* = .88) nor older (β = 0.40; 95% CI, −0.06 to 0.85; *P* = .09) female offspring showed a difference between risk groups. Younger (β = 0.30; 95% CI, −0.16 to 0.80; *P* = .21) and older (β = 0.10; 95% CI, −0.31 to 0.52; *P* = .65) male offspring retained their lack of difference between risk groups.

### Socioeconomic Status

**Figure 3.**
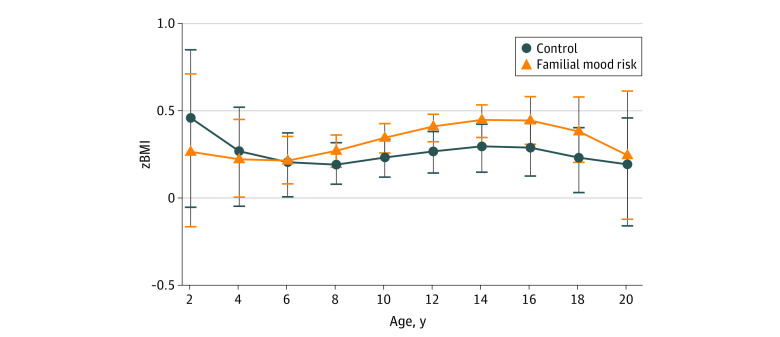
Association of Familial Mood Disorder Risk Status With Body Mass Indez *z* Score (*z*BMI) Accounting for Socioeconomic Status (SES) The association of familial mood disorder risk status on *z*BMI in male and female offspring as modeled by nonparametric regression estimates with SES as a covariate. Error bars represent the SEM at each 2-year age interval.

**Figure 4.**
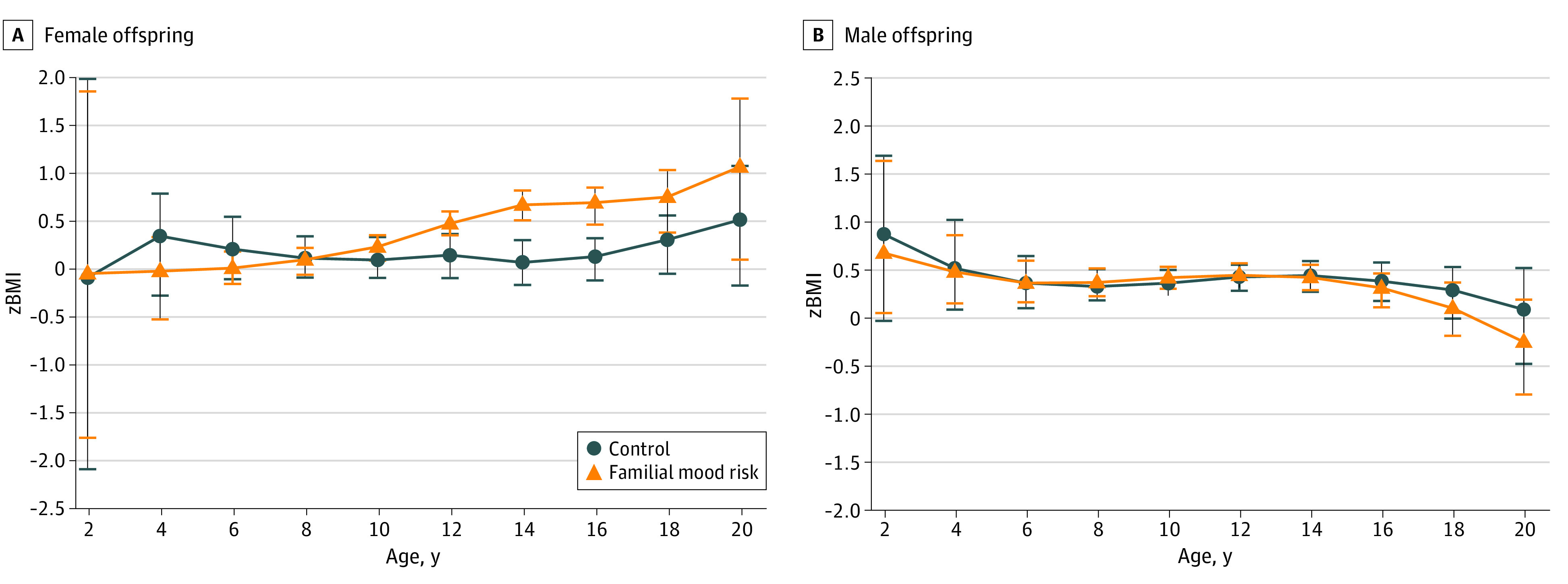
Association of Familial Mood Disorder Risk Status on Body Mass Index *z* Score (*z*BMI) Accounting for Socioeconomic Status (SES) by Sex The association of familial mood disorder risk status on *z*BMI in male and female offspring as modeled by nonparametric regression estimates with SES as a covariate. Error bars represent the SEM at each 2-year age interval.

When we added SES as a covariate, our results were unchanged from the primary analysis. In the full sample, individuals with familial mood risk showed no difference in *z*BMIs compared with controls (β = 0.11; 95% CI, −0.01 to 0.24; *P* = .06) (Figure 3). Female offspring with familial mood risk still had a higher *z*BMIs than controls (β = 0.25; 95% CI, 0.06-0.42; *P* = .01) (Figure 4A), driven by the significantly increased *z*BMIs in female offspring with familial mood risk at 12 years and older (β = 0.64; 95% CI, 0.3-0.91; *P* < .001), whereas younger female offspring showed no difference between groups (β = −0.07; 95% CI, −0.31 to 0.16; *P* = .57). Male offspring also showed the same trend as the primary model (Figure 4B). Neither younger (β = 0.05; 95% CI, −0.16 to 0.25; *P* = .67) nor older (β = −0.03; 95% CI, −0.29 to 0.22; *P* = .81) male offspring showed any difference in *z*BMI associated with risk status.

### Prematurity and Birth Weight

The final sensitivity analysis with prematurity status and birth weight showed the same results as the main analysis. Offspring of parents with mood disorders showed nominally higher *z*BMIs than controls (β = 0.14; 95% CI, 0.02-0.26; *P* = .03), driven by the significantly higher *z*BMIs in female offspring with familial mood risk at 12 years and older (β = 0.57; 95% CI, 0.30-0.84; *P* < .001). Marginal effects of prematurity and birth weight are presented in eTable 2 in Supplement 1.

## Discussion

This cohort study found that familial mood risk was associated with the trajectory of *z*BMI from childhood to early adulthood. From 12 years of age onward, female offspring with a family history of mood disorders had greater *z*BMIs than those without a family mood history. These differences are independent of socioeconomic status and early determinants of body mass, such as birth weight and prematurity status.

Sex differences in the prevalence of mood disorders have been well documented. Diagnoses of major depression are twice as common in female offspring from 12 years of age into adulthood, whereas rates of BD are similar between the sexes.^49,50^ Female individuals are also more likely to develop obesity than male individuals.^51^ This differential is mirrored in the sex differences noted in our study between weight trajectories in those with familial risk vs those without. Female offspring with familial mood risk who do not have a mood disorder themselves are still more likely to have a greater *z*BMI compared with those without familial risk, whereas male offspring do not differ in their likelihood of increased weight by familial mood risk status. It may be possible to identify an individual’s chance of developing mood problems and obesity in adulthood by considering their trajectory in adolescence.

To our knowledge, this is the first study to examine the differences in trajectory of body mass during childhood and adolescence between individuals with and without a family history of a mood disorder. Our results suggest that female offspring with familial risk for mood disorders are more likely to develop a higher weight in adolescence. These findings are consistent with studies^13,15^ in adults connecting mood disorder diagnosis with higher weight. Female adolescents are twice as likely to experience depression as male adolescents, and familial risk is the most significant variable.^52,53^ Typically, BD first presents with a depressive episode, mania, or hypomania appearing years later.^54^ Female adolescents seem to be developing physical health issues based on their mood disorder risk. Early identification of this risk allows us to pinpoint a window of opportunity for early intervention on a potentially modifiable risk factor that may provide a chance to decrease severity of future psychiatric illness.^14^

This study benefited from a cohort enriched with offspring of parents with mood disorders; 64.5% of the sample, much higher than the population mean, had parents with mood disorders.^55^ Affected parents transmit risk to their offspring via genetic and environmental factors, and a large body of research has shown the effectiveness of early intervention and prevention in youth with familial risk for mood disorders.^56,57^ If we identify and address risk early, there is a potential for implementing preventive measures.^58^ Early identification of individuals at risk of both physical health issues and mood disorders could inform future interventions.

### Future Directions

Weight of offspring may also have been affected by parental lifestyle choices, which may have been influenced by their disorder and its severity. This study did not consider lifestyle factors that could be associated with the developmental trajectory of body weight, including sleep quality, activity levels, or food habits, all which have been shown to affect mood disorder severity and body weight.^59,60,61,62^

We considered sex differences in this study; moving forward, it will be important to consider the effect of gender identity on these trends. Are the differences between youth with and without mood risk consistent with an individual’s sex or gender when the two are different? Transgender individuals have elevated rates of mood disorders, and it is possible they may be particularly vulnerable to physical health issues as a result.^63^

### Strengths and Limitations

A strength of this study is that the sample benefited from researcher-collected body measurements, avoiding the concern of self-report bias; men and women both overestimate their own height and men tend to overestimate weight, while women underestimate weight.^64^ As with all research, this study was limited in some ways. Ten percent of youth in the study had missing data on weight or height. Participants with missing data did not significantly differ in sex distribution but were younger, had lower SES, and had increased familial mood risk (eTable 1 in Supplement 1). Their younger age is expected because these participants had fewer opportunities to provide measurements. Individuals with serious mental illness, especially those with physical comorbidities, are known to have many barriers to study participation, such as ongoing symptoms and logistical concerns.^65^ Parents in the familial high-risk group are likely influenced by these factors, which could have led to the decreased participation of offspring. Additionally, decreased study participation and negative metabolic health outcomes are associated with lower SES, so our excluded group may be especially vulnerable to developing physical health issues.^66,67^ It will be important to try to obtain physical measurements from these participants in future assessments and compare them to the main sample.

## Conclusions

In this cohort study of youth at familial risk for mood disorders, we found that offspring of parents with mood disorders had increased *z*BMIs compared with controls with unaffected parents starting at approximately 12 years of age. This increase was driven by female adolescents. It is important to address risk factors in this group because they are particularly vulnerable to developing both mood and physical health issues. Adolescents face immense pressure related to body image and weight, and individuals at risk for depression are prone to being highly self-critical, so it will be vital to address this topic in a sensitive and compassionate way, especially in the context of intervention.^68,69^ We identified a period of development in which female adolescents with a family history of mood disorders began to show a significantly higher *z*BMI compared with controls. Further work should consider the association of early intervention on protecting against development of mood and weight conditions in this high-risk group. Additionally, education on the association between mental and physical health with thoughtful consideration of the stigma associated with mental health conditions and increased body weight would provide a great opportunity to empower youth to take charge of their own health.

## Notes

Supplement 1. **eMethods 1.** Waist-to-Height Ratio **eMethods 2.** Pubertal Status **eTable 1.** Demographic Characteristics of Participants With and Without Valid Weight and Height Data **eTable 2.** Model Coefficients for Each Level of Prematurity and Birth Weight in All Offspring and Older Females
eReferences

Supplement 2.
Data Sharing Statement
