# Supplementary material for: Developmental Trajectory of Body Weight in Youths at Risk for Major Mood Disorders
Source: JAMA Netw Open. 2023 Oct 19;6(10):e2338540. doi: 10.1001/jamanetworkopen.2023.38540 (PMC10587790; doi:10.1001/jamanetworkopen.2023.38540)
Supplement: Supplement 1. — eMethods 1. Waist-to-Height Ratio eMethods 2. Pubertal Status eTable 1. Demographic Characteristics of Participants With and Without Valid Weight and Height Data eTable 2. Model Coefficients for Each Level of Prematurity and Birth Weight in All Offspring and Older Females eReferences [file jamanetwopen-e2338540-s001.pdf]

Supplementary Online Content

Adepalli N, Cumby J, Campbell N, et al. Developmental trajectory of body weight in youth at risk for major mood disorders. *JAMA Netw Open*. 2023;6(10):e2338540. doi:10.1001/jamanetworkopen.2023.38540

eMethods 1. Waist-to-Height Ratio

eMethods 2. Pubertal Status

eTable 1. Demographic Characteristics of Participants With and Without Valid Weight and Height Data

eTable 2. Model Coefficients for Each Level of Prematurity and Birth Weight in All Offspring and Older Females

eReferences

This supplementary material has been provided by the authors to give readers additional information about their work.

© 2023 Adepalli N et al. *JAMA Network Open.*

eMethods 1. Waist-to-Height Ratio

Methods

Though BMI is the most commonly used measure of obesity, other measures that reflect body shape may be more informative of health risks as BMI can overestimate obesity.[1,2] Measures of central adiposity, including waist circumference, waist-to-hip ratio, and waist-to-height ratio (WtH) are better predictors of obesity-related cardiovascular risk than BMI.[3] WtH ratio correlates well with intra-abdominal fat[4], which is a stronger indicator of long-term morbidity and mortality than BMI which more closely describes peripheral fat distribution.[5] WtH ratio is also more accurate than BMI for measuring cardiometabolic risk in adults and children.[6,7] WtH ratio is elevated in children ages 9 – 10 with a history of depression, especially in females.[8] WtH ratios were converted to age- and sex- adjusted z scores (zWtH) using the NHANES III LMS tables provided by Sharma and colleagues.[9]

Results

In 878 measurements from 307 participants with zWtH available, we found that there was no difference in zWtH between individuals with high familial mood risk and controls (β = 0.10, 95% CI -0.01 to 0.23, p = 0.116).

In females, we found that those with high familial mood risk did not differ in zWtH from controls (β = 0.13, 95% CI -0.05 to 0.31, p = 0.155). In younger females, those with high familial mood risk did not show a difference in zWtH compared to controls (β = -0.04, 95% CI -0.33 to 0.24, *p* = 0.788), but in the older females, those with a family history of mood disorders showed a nominal increase in zWtH compared to controls (β = 0.24, 95% CI 0.02 to 0.48, *p* = 0.031).

In males, we found that those with high familial mood risk had no significant difference in zWtH compared to controls (β = 0.09, 95% CI -0.08 to 0.26, *p* = 0.289) in both the younger (β =

0.09, 95% CI -0.15 to 0.34, *p* = 0.486) and older age groups (β = 0.09, 95% CI -0.14 to 0.32, *p* = 0.455).

These results, though attenuated, support the findings from our analysis using zBMI as a metric for body composition; females over age 12 with familial mood risk show increased zWtH ratios compared to controls, while other groups did not differ by familial mood risk. WtH ratios are more representative of abdominal obesity particularly in youth, but BMI is used more widely in the literature and is useful for comparison to other studies.

Using one zWtH measurement per individual (n = 308), we found that the effect sizes of our results were decreased from the main zWtH results in older (β = 0.02, 95% CI -0.37 to 0.45, *p* = 0.918) and younger females (β = 0.05, 95% CI -0.39 to 0.65, p = 0.850) but were increased in younger (β = 0.50, 95% CI -0.06 to 1.13, *p* = 0.096) and older males (β = 0.22, 95% CI -0.13 to 0.56, p = 0.203).

When we ran sensitivity analyses with SES as a covariate, we found the same trends as the primary model. Older females with familial mood risk had increased zWtH compared to controls (β = 0.31, 95% CI 0.08 to 0.58, *p* = 0.017), while younger females (β = -0.08, 95% CI -0.34 to 0.27, *p* = 0.623) and all males (younger: β = 0.01, 95% CI -0.23 to 0.24, *p* = 0.917; older: β = 0.09, 95% CI -0.11 to 0.32, *p* = 0.419) in our sample showed no difference between individuals with and without mood risk.

Sensitivity analyses accounting for prematurity status and birth weight also showed the same trends as the primary model. Older females with familial mood risk had greater zWtH than controls (β = 0.24, 95% CI –0.01 to 0.49, *p* = 0.070) with similar effect size, while younger females (β = 0.03, 95% CI -0.32 to 0.37, *p* = 0.859) and all males showed no difference between

© 2023 Adepalli N et al. *JAMA Network Open.*

groups (younger: β = 0.06, 95% CI -0.20 to 0.32, $p = 0.673$; older: β = 0.08, 95% CI -0.17 to 0.28, $p = 0.507$).

The results of our zWtH analyses paralleled the pattern we found in zBMI; older females with familial mood risk had a significantly increased zWtH compared to controls, while younger females and males did not show any difference based on familial mood risk. In sensitivity analyses, we found results were largely unchanged from the main analysis.

© 2023 Adepalli N et al. *JAMA Network Open.*

eMethods 2. Pubertal Status

Methods

The observed rapid increase in zBMI in females around age 12 coincides with the typical period of pubertal onset. As such, we investigated the effect of using puberty onset as a cut-point instead in age-stratified analysis. Pubertal status was determined using the Growing and Changing Questionnaire (GCQ) in youth over age 8. This instrument is a combination of the Pearson's Puberty Development Scale (PDS)[10] and the Sexual Maturation Scale (SMS)[11,12] that provides a score from 1-5 for an individual. The PDS asks about body hair, skin changes, facial hair in males, and menarche in females. The SMS is made up of 5 illustrations that show advancing stages of secondary sex characteristics.

Sensitivity analyses were conducted for pubertal status as follows: participants were divided into pre- and post-pubertal groups by dichotomizing GCQ scores such that GCQs of 1 or 2 were considered pre-pubertal and scores of 3 to 5 were considered post-pubertal.[13]

Results

When offspring were divided by pubertal status rather than age 12, age-stratified results were unchanged from the primary analysis. Pre-pubertal females with familial mood risk did not differ in zBMI compared to controls (β = -0.04, 95% CI -0.30 to 0.21, p = 0.789). Post-pubertal females with a family history of mood disorders showed a significant increase in zBMI compared to controls (β = 0.36, 95% CI 0.13 to 0.54, p = 0.001). In pre-pubertal males, we found that those with high familial mood risk had no significant difference in zBMI compared to controls (β = 0.07, 95% CI -0.23 to 0.35, p = 0.353). This was the case with post-pubertal males as well (β = -0.12, 95% CI -0.36 to 0.10, p = 0.300).

All sensitivity analyses for SES and prematurity and birth weight mirrored the primary results. For repeated measures, post-pubertal females no longer had a significantly higher zBMI than controls, but effect size was comparable to main results ($\beta = 0.36$, 95% CI -0.02 to 0.75, $p = 0.096$).

The similarity of these results to the primary findings suggests that pubertal timing is not greatly affecting the patterns seen in zBMI development between at-risk youth and controls. Future work could consider the trends of zBMI over pubertal development between youth at familial mood risk and controls.

© 2023 Adepalli N et al. *JAMA Network Open.*

eTable 1. Demographic Characteristics of Participants With and Without Valid Weight and Height Data

Differences between groups were tested using chi-square (x^2) for categorical variables and t-test for continuous variables. * denotes statistically significant differences between groups at p<0.05.

	**Included in analysis (n = 394)**	**Missing height/weight data (n = 43)**	**p-value**
**Number of individuals at high familial risk (%)***	254 (64.5%)	23 (53.5%)	0.0312
**Female participants (%)**	191 (48.5%)	21 (48.8%)	0.629
**Male participants (%)**	203 (51.5%)	22 (51.2%)	
**SES, number of assessments by category (%)**			0.069
**0**	17 (4.3%)	3 (7.0%)	
**1**	38 (9.6%)	9 (20.9%)	
**2**	59 (15.0%)	3 (7.0%)	
**3**	107 (27.2%)	16 (37.2%)	
**4**	147 (37.3%)	9 (20.9%)	
**5**	60 (15.2%)	3 (7.0%)	
**Age, mean (SD)***	11.5 (3.6)	6.5 (4.1)	2.2×10^{-16}

© 2023 Adepalli N et al. *JAMA Network Open.*

eTable 2. Model Coefficients for Each Level of Prematurity and Birth Weight in All Offspring and Older Females

**All offspring**			
	**Beta**	**95% CI**	**p-value**
Prematurity			
Extremely premature-very premature	-0.19	-0.68 to 0.18	0.399
Extremely premature-moderately premature	0.29	0.02 to 0.41	0.004
Extremely premature-Full term/overdue	-0.36	-0.54 to -0.21	<0.001
Birth weight			
Low-average	-0.19	-0.30 to -0.08	<0.001
Low-large	-0.58	-0.81 to -0.36	<0.001
**Females over age 12**			
	**Beta**	**95% CI**	**P value**
Prematurity			
Extremely premature-very premature	-0.41	-0.73 to -0.40	0.026
Extremely premature-Moderately premature	-	-	-
Extremely premature-Full term/overdue	-0.05	-0.35 to 0.24	0.745
Birth weight			
Low-average	-0.37	-0.65 to -0.10	0.006
Low-large	-0.79	-0.17 to -0.27	0.002

© 2023 Adepalli N et al. *JAMA Network Open.*

eReferences

1. Charbonneau-Roberts G, Saudny-Unterberger H, Kuhnlein HV, Egeland GM. Body mass index may overestimate the prevalence of overweight and obesity among the Inuit. *Int J Circumpolar Health*. 2005;64(2):163-169.

2. Kaluski DN, Keinan-Boker L, Stern F, et al. BMI may overestimate the prevalence of obesity among women of lower socioeconomic status. *Obesity (Silver Spring)*. 2007;15(7):1808-1815.

3. Lee CM, Huxley RR, Wildman RP, Woodward M. Indices of abdominal obesity are better discriminators of cardiovascular risk factors than BMI: a meta-analysis. *J Clin Epidemiol*. 2008;61(7):646-653.

4. Ashwell M, Cole TJ, Dixon AK. Ratio of waist circumference to height is strong predictor of intra-abdominal fat. *BMJ*. 1996;313(7056):559-560.

5. Bigaard J, Frederiksen K, Tjønneland A, et al. Waist circumference and body composition in relation to all-cause mortality in middle-aged men and women. *Int J Obes (Lond)*. 2005;29(7):778-784.

6. Ashwell M, Gunn P, Gibson S. Waist-to-height ratio is a better screening tool than waist circumference and BMI for adult cardiometabolic risk factors: systematic review and meta-analysis. *Obes Rev*. 2012;13(3):275-286.

7. Lo K, Wong M, Khalechelvam P, Tam W. Waist-to-height ratio, body mass index and waist circumference for screening paediatric cardio-metabolic risk factors: a meta-analysis. *Obes Rev*. 2016;17(12):1258-1275.

8. Lewis-de Los Angeles WW, Liu RT. History of Depression, Elevated Body Mass Index, and Waist-to-Height Ratio in Preadolescent Children. *Psychosom Med*. 2021;83(9):1075-1081.

9. Lewis-de Los Angeles WW, Liu RT. History of Depression, Elevated Body Mass Index, and Waist-to-Height Ratio in Preadolescent Children. *Psychosom Med*. 2021;83(9):1075-1081.

10. Carskadon, M. A., & Acebo, C. (1993). A self-administered rating scale for pubertal development. *Journal of Adolescent Health*, *14*(3), 190-195.

11. Marshall, W. A., & Tanner, J. M. (1970). Variations in the pattern of pubertal changes in boys. *Archives of disease in childhood*, *45*(239), 13-23.

12. Marshall, W. A., & Tanner, J. M. (1969). Variations in pattern of pubertal changes in girls. *Archives of disease in childhood*, *44*(235), 291.

13. Wescott, D. L., Morash-Conway, J., Zwicker, A., Cumby, J., Uher, R., & Rusak, B. (2019). Sleep in offspring of parents with mood disorders. *Frontiers in Psychiatry*, *10*, 225.

© 2023 Adepalli N et al. *JAMA Network Open.*
