# Supplementary material for: Developmental Trajectory of Body Weight in Youths at Risk for Major Mood Disorders
Source: JAMA Netw Open. 2023 Oct 19;6(10):e2338540. doi: 10.1001/jamanetworkopen.2023.38540 (PMC10587790; doi:10.1001/jamanetworkopen.2023.38540)
Supplement: Supplement 2. — Data Sharing Statement [file jamanetwopen-e2338540-s002.pdf]

# Data Sharing Statement

Adepalli. Developmental Trajectory of Body Weight in Youths at Risk for Major Mood Disorders. *JAMA Netw Open*. Published October 19, 2023.
doi:10.1001/jamanetworkopen.2023.38540

**Data**
**Data available:** No
